# Serum Chemokine CXCL7 as a Diagnostic Biomarker for Colorectal Cancer

**DOI:** 10.3389/fonc.2019.00921

**Published:** 2019-10-09

**Authors:** Longhai Li, Lihua Zhang, Yu Tian, Ting Zhang, Guangliang Duan, Yankui Liu, Yuan Yin, Dong Hua, Xiaowei Qi, Yong Mao

**Affiliations:** ^1^Department of Pathology, Affiliated Hospital of Jiangnan University, Wuxi, China; ^2^School of Pharmacy, Jiangnan University, Wuxi, China; ^3^Wuxi Oncology Institute, Affiliated Hospital of Jiangnan University, Wuxi, China; ^4^Department of Oncology, Affiliated Hospital of Hangzhou Normal University, Hangzhou, China; ^5^Department of Oncology, Affiliated Hospital of Jiangnan University, Wuxi, China

**Keywords:** cancer biomarker, colorectal cancer, CXCL7, diagnosis, serum

## Abstract

Identification of effective biomarkers is crucial for monitoring the treatment and remission of colorectal cancer (CRC) and improving survival. It is particularly important to diagnose CRC before the tumor metastasizes (stage I–II disease) where possible, to provide the greatest opportunity for patient recovery. Here, we evaluated the clinical value of serum chemokine (C-X-C) ligand 7 (CXCL7) concentration as a biomarker for CRC diagnosis. An enzyme-linked immunosorbent assay was used to measure CXCL7 concentration in 560 serum samples from patients with CRC and controls. Logistic regression and receiver operating characteristic (ROC) curve analysis was used to assess the diagnostic efficacy and build mathematical diagnostic models. The concentration of CXCL7 in the CRC group was significantly higher than that in the control group (*P* < 0.001), with an area under the ROC curve (AUC) value of 0.862 [95% confidence interval (CI): 0.831–0.890]. Further, the AUC of a regression model including the markers carcinoembryonic antigen (CEA), carbohydrate antigen 19-9 (CA19-9), and carbohydrate antigen 125 (CA125), along with CXCL7, was 0.933 (95% CI: 0.909–0.952). For stage I–II tumors, CXCL7 had the highest AUC (0.823, 95% CI: 0.783–0.858) among the four individual biomarkers. The AUC value for combination model analysis of samples from patients with stage I–II tumors was 0.904 (95% CI: 0.872–0.930), with a sensitivity of 82.76% and a specificity of 87.14%, and an optimal cut-off value of 2.66. AUC values for application of the regression model in subgroup analysis were 0.947 (0.917–0.968) and 0.919 (0.874–0.951) for males and females, respectively. These results suggest that CXCL7 has potential as a serum diagnostic biomarker for detection of CRC. Importantly, the combination of CXCL7, CEA, CA125, and CA19-9 may facilitate diagnosis of CRC with relatively high sensitivity and specificity.

**Clinical Trial Registration Number:** LS2017001.

## Introduction

Colorectal cancer (CRC) is a common malignant tumor with high levels of mortality (1); it is the third most common cancer and the fourth leading cause of cancer-related deaths worldwide (2). Further, CRC incidence and mortality are rising annually, with a person presenting an average risk for CRC having a 5% chance of developing the disease (3, 4). When diagnosed before metastasis, CRC patients have a favorable clinical prognosis, with a 5-year survival rate of 70% to 90% (5); however, if the tumor has metastasized, patients often have poor prognoses, with 5-year survival rates of <20%, even after surgery and comprehensive postoperative treatment (6).

Therefore, it is critical to detect CRC before it metastasizes, to enable timely and appropriate treatment and prevent disease progression. Currently, colonoscopy and tissue biopsy are the most effective methods of CRC diagnosis; however, colonoscopy is an invasive procedure that can cause trauma to patients, and the entire surgical process can sometimes be difficult to complete owing to poor compliance among patients with CRC (7). Furthermore, given the invasiveness and expense of these procedures, the implementation of universal screening as part of routine physical examination is impractical (8). Hence, more non-invasive, sensitive, and effective biomarkers are urgently needed for clinical application. As collection of peripheral blood is a relatively simple, painless, and non-invasive procedure, and blood is convenient to store and process for analysis, there has been a focus on identification of blood biomarkers in recent years (9). Carcinoembryonic antigen (CEA), carbohydrate antigen 19-9 (CA19-9), and carbohydrate antigen 125 (CA125) are widely used in tumor detection (10); however, these three biomarkers, alone or in combination, are inadequate for CRC diagnosis, owing to their low sensitivity and specificity (11). Hence, it is imperative to identify additional effective serum biomarkers to facilitate optimization of the diagnosis and treatment of CRC, particularly at non-metastatic stages (stages I–II).

Chemokine (C-X-C) ligand 7 (CXCL7) is a platelet-derived growth factor belonging to the CXC family of chemokines (12, 13). CXCL7 has an important role in tumorigenesis and is associated with the proliferation and metastasis of various tumors (14). Immunohistochemistry, polymerase chain reaction, and other techniques have been used to elucidate relationships among tumor tissues, gene expression, and protein levels (15). In this study, we quantitatively analyzed CXCL7 levels in 560 serum samples using an enzyme-linked immunosorbent assay (ELISA). In addition, we selected common tumor-associated antigen indicators (CEA, CA125, and CA19-9) for combination diagnostic testing, and used receiver operating characteristic (ROC) curves (16) to investigate the diagnostic efficiency of CXCL7 alone or combined with these common biomarkers.

In this study, we explored the usefulness of CXCL7 as a new diagnostic biomarker for CRC. CXCL7 levels were higher in patients with CRC relative to controls [the statistical differences between CRC patients and controls for other chemokines in serum samples were less notable (data not shown)], consistent with our hypothesis that serum CXCL7 levels are elevated in patients with CRC. Our analysis also demonstrates that serum CXCL7 is a potential biomarker for the diagnosis of CRC.

## Materials and Methods

### Patients and Samples

A total of 560 subjects were recruited from the Affiliated Hospital of Jiangnan University, China, from January 2016 to August 2019, comprising 280 biopsy-proven patients with CRC and 280 controls. The selection criteria were as follows. (1) CRC was diagnosed based on pathology results, and diagnosis and clinical stage were determined using the standards published by the American Joint Committee on Cancer and the Union for International Cancer Control. (2) CRC was first diagnosed within the study period. (3) Patients had CRC only; those with multiple tumors or possible metastasis were excluded. (4) The CRC group did not receive chemotherapy, radiotherapy, or surgery before blood samples were collected. (5) Patients with CRC had no hematological disorders, serious digestive system abnormalities, infectious diseases, liver or kidney malfunctions, or immune system defects. (6) Patients with CRC had no history of surgical treatment in the previous 2 years. Controls were selected at a health checkup center, matched by age and sex, and did not have malignant disease, cardiovascular disease, or any other serious illness. All subjects provided written informed consent, and all procedures were approved by the Hospital Ethics Committee.

To determine the diagnostic value of serum CXCL7 for CRC, the 560 subjects were first randomly assigned into training and validation sets. There were 266 subjects in the training set (133 CRC patients and 133 controls) and 294 in the validation set (147 CRC patients and 147 controls). The training set was used to explore the potential utility of the biomarker, and the validation set was used to verify its diagnostic efficacy. Cross-validation was conducted using all subjects in the training set (set A: patients with CRC were designated “Ap” and controls “Ac”) and in the validation set (set B: patients with CRC were designated “Bp” and controls “Bc”). All 280 patients with CRC were included in the analysis to explore the diagnostic efficiency of CXCL7.

Blood samples were collected before any surgical procedures, then centrifuged at 2,000 × g for 20 min to obtain serum. An aliquot of each serum sample was sent to the clinical laboratory for measurement of CEA, CA125, and CA19-9 levels. The remaining serum samples were stored at −80°C for chemokine testing.

### Enzyme-Linked Immunosorbent Assay

Serum CXCL7 expression was measured in samples from the 560 subjects using the commercial human neutrophil-activating peptide 2 (NAP-2/CXCL7) ELISA Kit (Shanghai Langdon Biotechnology; Shanghai, China), according to the manufacturer's instructions. ELISA kits and serum samples were equilibrated to room temperature for 30 min prior to the assay. Subsequently, 50 μl serum samples were added to each well in the 96-well plate, followed by 50 μl of biotin antigen working fluid. Then, plates were incubated at 37°C for 30 min and washed five times with buffer, and an affinity-horseradish peroxidase (HRP) antibody was added for a second incubation. Subsequently, after washing for a second time, chromogenic solutions A and B were added to the assay and incubated for 10 min, followed by a terminating solution. Finally, samples were analyzed using a full-wavelength microplate reader at 450 nm (MD VersaMax, Molecular Devices; California, USA) to determine absorbance values. Levels of CEA, CA125, and CA19-9 were quantified using a chemiluminescent immunoassay (Roche Diagnostics GmbH, Mannheim, Germany). All the CXCL7 measurements were conducted in duplicate. CEA, CA125, and CA19-9 concentrations were normalized to those of CXCL7.

### Statistical Analysis

SPSS 20.0 statistical software (IBM; Armonk, NY, USA) was used for statistical analyses. Nonparametric statistical methods were used, unless otherwise stated. The median (M) and interquartile range (IQR; Q_1_-Q_3_) were used to describe continuous variables, with frequencies and percentages (%) used for categorical variables. The difference in serum levels between two groups were evaluated by Mann-Whitney *U*-test, and the Kruskal-Wallis H-test was applied for comparisons of three or more groups. The chi-square (χ^2^) test was used for categorical variables. Analysis of correlation between variables was assessed using Spearman's rank correlation. ROC curve analysis was used to determine the diagnostic value of serum biomarker expression in patients with CRC. Other diagnostic parameters were also evaluated, including sensitivity, specificity, cut-off value, positive predictive value, negative predictive value, and area under the ROC curve (AUC) with 95% confidence interval (CI), to assess the discrimination power of individual or combined biomarkers.

Univariate and multivariate logistic regression analyses were adopted to assess the strength of associations between risk factors and CRC, and curves were generated to determine optimal cut-off values. All processes were normalized using CXCL7 levels, and a two-sided *P* < 0.05 was considered statistically significant. *P*-values were corrected (corrected *P* = 0.0083) for multiple testing using the Bonferroni test.

## Results

### Levels of CXCL7 and Tumor Associated Antigens (CEA, CA125, and CA19-9) in Patients With CRC and Controls

**Figure 1:**
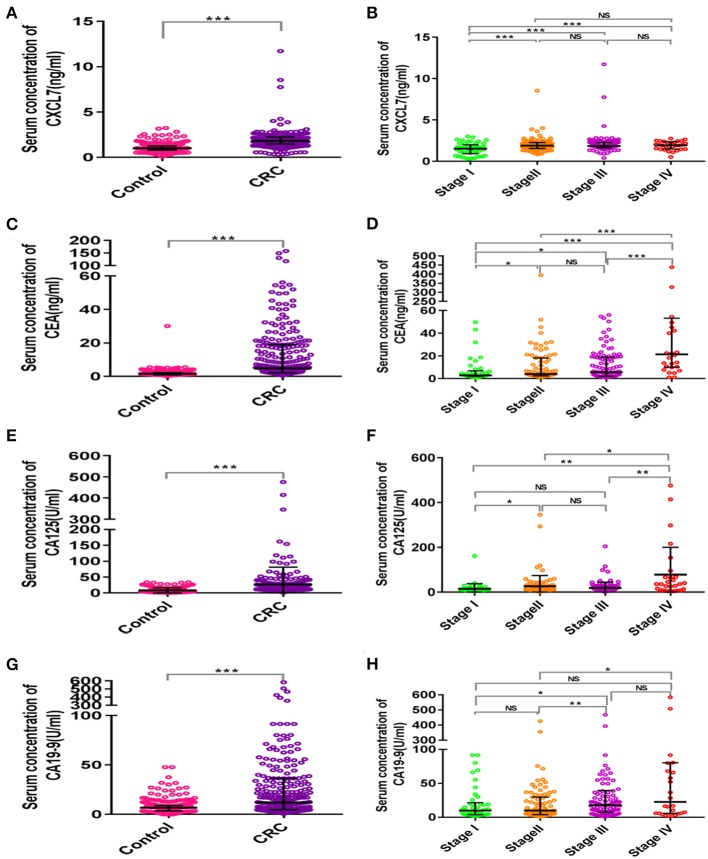
Scatter plot analysis of serum concentrations of CXCL7 and tumor-associated antigens in patients with CRC and controls. **(A)** Comparison of serum CXCL7 concentrations between the control and CRC groups. **(B)** Comparison of serum CXCL7 concentrations among different TNM subgroups. **(C–H)** Comparisons of serum CEA, CA125, and CA19-9 levels between the control and CRC groups and comparisons among different TNM subgroups. The Mann-Whitney U-test was used for comparisons between two groups and the Kruskal-Wallis test was applied for analysis of three or more groups. *P < 0.05, **P < 0.01, ***P < 0.001.

The average age of the 280 patients with CRC was 62.30 ± 9.73 years; 166 were male and 114 female. The mean age of the control group was 61.26 ± 5.80 years; 178 were male and 102 female. CRC patients and controls were comparable in terms of age and sex (both *P* > 0.05). Moreover, the median concentration of CXCL7 in the CRC group was 1.82 (IQR: 1.49–2.23) ng/ml, significantly higher than that in the control group (M = 1.02 ng/ml, IQR: 0.81–1.27; *P* < 0.001; Figure 1A). In addition, median serum CXCL7 concentrations in samples from patient with tumors at each TNM (tumor-node-metastasis) stage were all higher than that of the control group (all *P* < 0.001; Figure 1B). Similarly, patients with CRC had higher serum CEA, CA125, and CA19-9 levels than controls (all *P* < 0.05; Figures 1C–H; Supplementary Table 1).

### Correlation Between CXCL7 Level and Clinical Characteristics in Patients With CRC

Correlations between CXCL7 level and clinical characteristics of all 280 patients with CRC were analyzed. Serum CXCL7 expression was significantly associated with sex (r = −0.143; *P* = 0.016), TNM stage (r = 0.185; *P* = 0.002), and T stage (r = 0.264; *P* < 0.001). No significant associations were found between serum CXCL7 levels and other pathological parameters (Table 1).

**Table 1:** Correlation between CXCL7 level and clinical characteristics in patients with CRC.

**Variable**		**Number**	**Median**	**IQR**	**r**	***P*-value**
Age (years)	<60	103	1.78	1.46–2.16	0.075	0.211
	≥ 60	177	1.89	1.51–2.27		
Sex	Male	166	1.92	1.52–2.29	−0.143	0.016
	Female	114	1.76	1.46–2.15		
Tumer size	<4 cm	171	1.82	1.50–2.28	−0.037	0.542
	≥4 cm	109	1.82	1.48–2.19		
Histological grade	Well	14	1.69	1.48–2.13	−0.012	0.836
	Moderately	199	1.84	1.53–2.22		
	Poor	67	1.81	1.38–2.28		
Location	Right	215	1.81	1.49–2.23	0.017	0.781
	Left	65	1.93	1.48–2.24		
TNM stage	I	50	1.52	0.91–1.99	0.185	0.002
	II	95	1.89	1.56–2.27		
	III	106	1.85	1.63–2.25		
	IV	29	1.93	1.48–2.35		
T stage	1–2	81	1.63	1.21–2.10	0.264	<0.001
	3–4	199	1.90	1.63–2.29		
N stage	0	151	1.77	1.39–2.21	0.112	0.060
	1–2	129	1.88	1.57–2.28		
M stage	0	251	1.81	1.50–2.21	0.047	0.431
	1	29	1.93	1.48–2.29		

### Diagnostic Value of CXCL7 in the Training and Validation Sets

**Figure 2:**
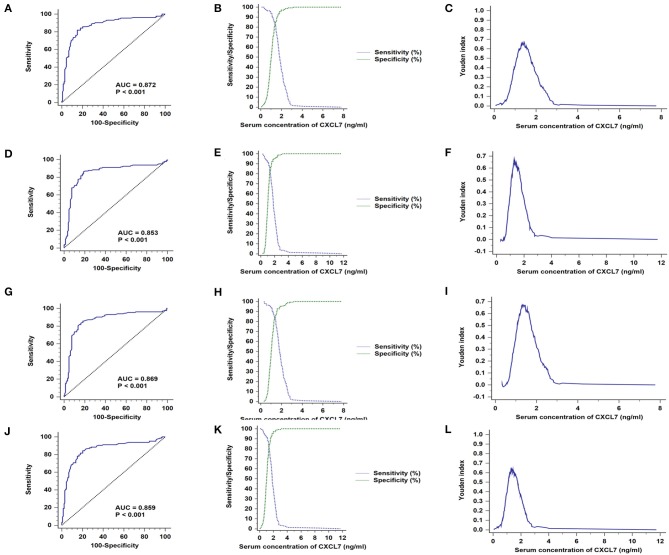
CXCL7 diagnostic test in training and validation sets. **(A)** AUC in the training set. **(B)** Sensitivity and specificity in relation to serum CXCL7 concentration in the training set. **(C)** Youden index in the training set. **(D)** AUC in the validation set. **(E)** Sensitivity and specificity in the validation set. **(F)** Youden index in the validation set. **(G–L)** Cross-validation ROC analyses.

The general clinical characteristics of the training and validation sets are presented in Supplementary Table 2. In both sets, serum CXCL7 expression was significantly higher in patients with CRC than in controls (both *P* < 0.05; Supplementary Table 3). ROC curve analysis indicated that serum CXCL7 expression is a potential diagnostic biomarker for CRC, with AUC values of 0.872 (training set, Figure 2A) and 0.853 (validation set, Figure 2D). In the cross-validation sets, AUC values were 0.869 (Ap/Bc, *P* < 0.05; Figure 2G) and 0.859 (Bp/Ac, *P* < 0.05; Figure 2J). Sensitivity, specificity, and cut-off values are shown in Figures 2B,C,E,F,H,I,K,L; Supplementary Table 3.

### Diagnostic Efficiency of Serum CXCL7 in Samples From Patients With Different TNM Stage Tumors

**Figure 3:**
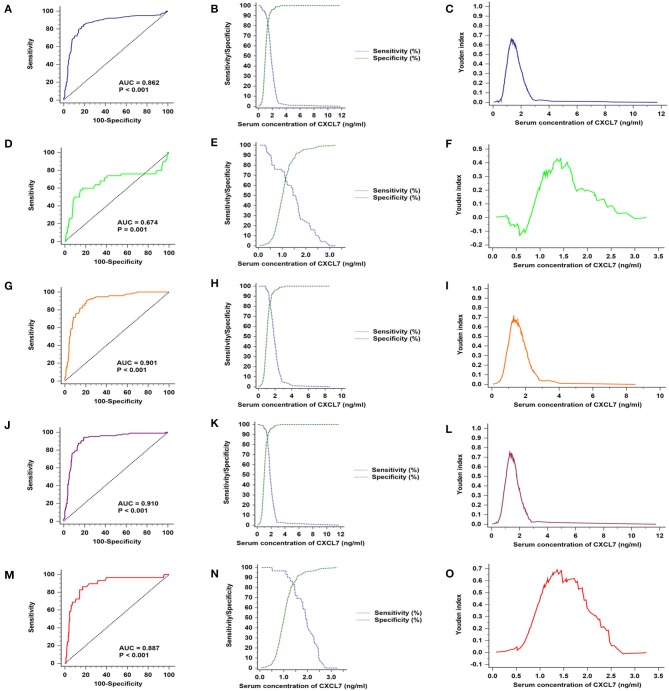
Serum CXCL7 ROC curves for samples from patients with different TNM stage tumors. **(A)** ROC curves for all patients with CRC and control subjects. **(B)** Sensitivity and specificity in relation to serum CXCL7 concentration. **(C)** Youden index in relation to serum CXCL7 concentration. **(D–O)** Diagnostic tests for tumor stages I to IV.

Based on data from all 280 patients with CRC and 280 controls, the AUC value for serum CXCL7 level was 0.862 (95% CI: 0.831–0.890; *P* < 0.001; Figure 3A). The cut-off value was 1.30 ng/ml, at the highest Youden index (sensitivity + specificity – 1; 0.6571; Figure 3C), with a sensitivity of 85.00% and specificity of 80.71% (Figure 3B; Table 2). For tumor stages I–IV, the AUC values for ROC curve analysis, based on serum CXCL7, were 0.674 (0.621–0.725; *P* < 0.001), 0.901 (0.866–0.929; *P* < 0.001), 0.910 (0.877–0.937; *P* < 0.001), and 0.887 (0.846–0.920; *P* < 0.001), respectively (Figures 3D,G,J,M). The sensitivities and specificities of CXCL7 levels for diagnosis of tumors at stages I–IV are presented in Figures 3E,H,K,N; Table 2; cut-off values are shown in Figures 3F,I,L,O.

**Table 2:** Results of the diagnostic tests performed between the CRC patients and the controls.

**Stages**	**Markers**	**Cutoff**	**AUC (95%CI)**	**Sensitivity (%)**	**Specificity (%)**	**PPV (%)**	**NPV (%)**
All CRC	CXCL7	1.30	0.862 (0.831–0.890)	85.00	80.71	81.51	84.33
	CEA	2.46	0.834 (0.800–0.863)	71.07	82.14	79.92	73.95
	CA125	6.45	0.749 (0.711–0.785)	85.71	61.79	69.16	81.22
	CA19-9	15.14	0.697 (0.657–0.735)	46.43	92.50	86.09	63.33
	Combination	2.50	0.933 (0.909–0.952)	87.14	87.50	87.50	87.50
Stage I–II	CXCL7	1.28	0.823 (0.783–0.858)	80.00	78.93	65.91	88.35
	CEA	2.46	0.818 (0.778–0.854)	68.97	82.14	66.67	83.64
	CA125	6.45	0.746 (0.702–0.787)	87.59	61.79	54.27	90.58
	CA19-9	9.10	0.632 (0.585–0.678)	52.41	77.14	54.29	75.79
	Combination	2.66	0.904 (0.872–0.930)	82.76	87.14	76.43	90.67
Male subgroup	CXCL7	1.30	0.909 (0.874–0.937)	87.95	85.39	84.88	88.37
	CEA	2.51	0.817 (0.772–0.857)	70.48	83.15	79.59	75.31
	CA125	6.45	0.766 (0.718–0.810)	84.34	67.98	71.07	82.31
	CA19-9	9.76	0.692 (0.640–0.740)	59.04	79.78	73.13	67.62
	Combination	2.13	0.947 (0.917–0.968)	86.14	94.96	94.08	88.02
Female subgroup	CXCL7	1.43	0.783 (0.722–0.836)	78.07	78.43	80.18	76.19
	CEA	1.98	0.860 (0.806–0.903)	84.21	75.49	79.34	81.05
	CA125	6.30	0.724 (0.660–0.783)	89.47	50.00	66.67	80.95
	CA19-9	16.77	0.706 (0.640–0.766)	47.37	96.08	93.10	62.03
	Combination	4.63	0.919 (0.874–0.951)	72.81	98.04	96.51	76.15

### Correlation Between CXCL7 and Tumor-Associated Antigen Levels in the CRC Group

Correlations between CXCL7 levels and those of tumor-associated antigens (CEA, CA125, and CA19-9) were explored in patients with CRC. The only statistically significant correlation was between serum CXCL7 and CA19-9 in stage IV tumors. CXCL7 was not significantly correlated with CEA, CA125, or CA19-9 (all *P* > 0.05, Supplementary Figure 1) in the total CRC patient group nor in any TNM subgroup.

### ROC Analyses of CXCL7, CEA, CA125, and CA19-9, and Construction of Diagnostic Models for CRC

**Figure 4:**
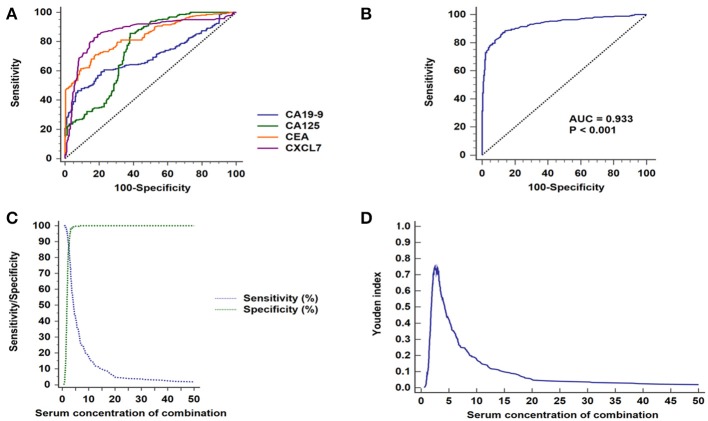
ROC curve analyses of CXCL7, CEA, CA125, and CA19-9 in patients with CRC. **(A)** ROC curves for the four individual biomarkers in patients with CRC. **(B)** ROC curves for the four biomarkers combined. **(C)** Sensitivity and specificity of the combined diagnostic test. **(D)** Youden index for the combined diagnosis test.

Among the four serum markers, CXCL7 had the highest AUC value (0.862, 95% CI: 0.831–0.890, *P* < 0.001) for CRC diagnosis, followed by CEA, CA125, and CA19-9 in that order (Figure 4A; Table 2). Logistic regression was used to build a diagnostic model that could explore whether combinations of biomarkers could improve the diagnostic efficacy. The resulting regression model was as follows:

Login (*P*) = −5.591 + 2.156 × CXCL7 + 0.314 × CEA + 0.062 × CA125 + 0.057 × CA19-9

The combination of the four biomarkers resulted in a superior diagnostic efficacy for patients with CRC (AUC = 0.932, 95% CI: 0.908–0.952; *P* < 0.001; Figure 4B), which was higher than the AUC values for each biomarker alone. Details are presented in Figure 4; Tables 2, 3.

**Table 3:** Regression model analysis for diagnosis testing.

**Stages**	**Variable**	**Univariate analysis**			**Multiivariate analysis**		
		**B**	**OR (95%CI)**	***P-*value**	**B**	**OR (95%CI)**	***P-*value**
All CRC	CXCL7	2.763	15.850 (9.957–25.231)	<0.001	2.156	8.637 (5.198–14.352)	<0.001
	CEA	0.480	1.616 (1.420–1.840)	<0.001	0.314	1.369 (1.192–1.573)	<0.001
	CA125	0.078	1.081 (1.058–1.104)	<0.001	0.062	1.064 (1.032–1.097)	<0.001
	CA19-9	0.072	1.074 (1.053–1.096)	<0.001	0.057	1.059 (1.031–1.087)	<0.001
Stage I–II	CXCL7	2.234	9.333 (5.790–15.044)	<0.001	1.796	6.824 (3.570–10.166)	<0.001
	CEA	0.453	1.572 (1.351–1.831)	<0.001	0.309	1.363 (1.164–1.595)	<0.001
	CA125	0.075	1.077 (1.052–1.103)	<0.001	0.065	1.067 (1.003–1.101)	<0.001
	CA19-9	0.061	1.063 (1.040–1.086)	<0.001	0.054	1.056 (1.026–1.086)	<0.001
Male subgroup	CXCL7	3.875	48.164 (21.716–106.825)	<0.001	3.206	24.676 (10.471–58.151)	<0.001
	CEA	0.542	1.720 (1.438–2.056)	<0.001	0.360	1.433 (1.122–1.830)	0.004
	CA125	0.078	1.081 (1.053–1.111)	<0.001	0.056	1.058 (1.013–1.105)	0.011
	CA19-9	0.066	1.068 (1.043–1.094)	<0.001	0.040	1.041 (1.006–1.077)	0.020
Female subgroup	CXCL7	1.711	5.533 (3.097–9.883)	<0.001	1.158	3.185 (1.614–6.285)	0.001
	CEA	0.405	1.499 (1.256–1.789)	<0.001	0.296	1.345 (1.146–1.578)	<0.001
	CA125	0.076	1.079 (1.042–1.116)	<0.001	0.071	1.075 (1.026–1.12)	0.002
	CA19-9	0.083	1.087 (1.047–1.128)	<0.001	0.084	1.088 (1.036–1.142)	0.001

### Diagnostic Efficacy of Biomarkers for Patients With CRC Prior to Metastasis

**Figure 5:**
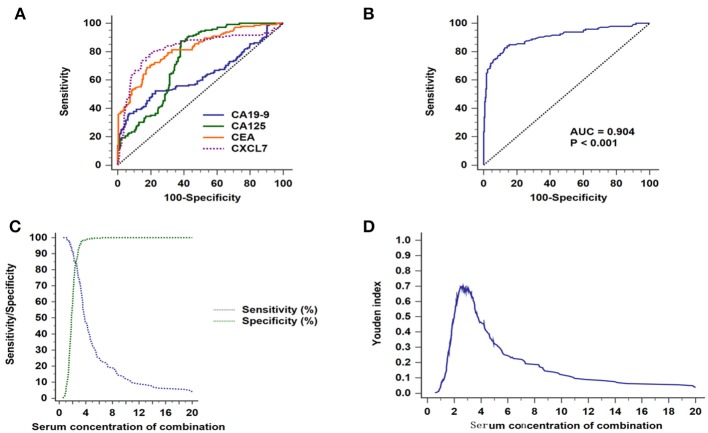
Diagnostic efficacy of biomarkers for CRC in patients with non-metastatic tumors. **(A)** ROC curves for the four individual biomarkers in patients with non-metastatic CRC. **(B)** ROC curves for the four combined biomarkers. **(C)** Sensitivity and specificity of the combined diagnostic test. **(D)** Youden index for the combined diagnostic test.

Patients with non-metastasized tumors (stage I–II) were selected for ROC analysis. Comparisons of individual biomarkers indicated that CXCL7 had the highest AUC value (0.823, 95% CI: 0.783–0.858; *P* < 0.001; Figure 5A; Table 2), with AUC values for CEA, CA125, and CA19-9 of 0.818 (95% CI: 0.778–0.854; *P* < 0.001), 0.746 (95% CI: 0.702–0.787, *P* < 0.001), and 0.632 (95% CI: 0.585–0.678, *P* < 0.001), respectively. The sensitivities and specificities at the optimal cut-off values for CXCL7 (1.28 ng/ml), CEA (2.46 ng/ml), CA125 (6.45 U/ml), and CA19-9 (9.10 U/ml) are detailed in Table 2. When the four biomarkers were combined, the resulting regression model was as follows:

Login (*P*) = −5.516 + 1.796 × CXCL7 + 0.309 × CEA + 0.065 × CA125 + 0.054 × CA19-9.

The AUC value for the combined analysis was 0.904 (95% CI: 0.872–0.930; *P* < 0.001; Figure 5B), with sensitivity and specificity of 82.76 and 87.14% (Figure 5C), respectively, and an optimal cut-off value of 2.66 (Figure 5D; Table 2).

### Analysis of Diagnostic Testing, According to Sex Subgroup

**Figure 6:**
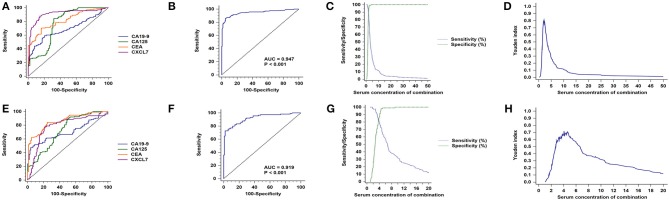
Sex subgroup analysis for diagnostic differentiation between patients with CRC and controls. **(A)** ROC curves for each of the four biomarkers in the male subgroup. **(B)** ROC curve for the four biomarkers combined in the male subgroup. **(C)** Sensitivity and specificity of the combined diagnostic test in the male subgroup. **(D)** Youden index for the combined diagnostic test in the male subgroup. **(E–H)** Equivalent results for diagnostic tests in the female subgroup.

As shown in Table 2, serum CXCL7 concentration differed between the male and the female subgroups; therefore, we conducted a subgroup analysis for diagnostic testing in these two subgroups separately. In the male group, among the four biomarkers tested individually, CXCL7 had the highest AUC value (0.909, 95% CI: 0.874–0.937; *P* < 0.001; Figure 6A). The logistic regression model was expressed as:

Login (*P*) = −6.816 + 3.206 × CXCL7 + 0.360 × CEA + 0.056 × CA125 + 0.040 × CA19-9.

The AUC value for the four biomarkers combined was 0.947 (95% CI: 0.917–0.968; *P* < 0.001, Figure 6B), with sensitivity and specificity of 86.14 and 94.96%, respectively (Figure 6C), and an optimal cut-off value of 2.13 (Figure 6D; Table 2). Similarly, CXCL7 combined with the other biomarkers had the highest AUC value for females (0.919, 95% CI: 0.874–0.951; *P* < 0.001; Figures 6E,F), with the logistic regression model:

Login (*P*) = −4.657 + 1.158 × CXCL7 + 0.296 × CEA + 0.073 × CA125 + 0.084 × CA19-9.

Sensitivity and specificity values are presented in Figures 6G,H; Table 2.

## Discussion

Malignant tumors have high mortality rates and a considerable economic and social impact on societies. Approximately 50% of patients with cancer live with mental and physical disabilities as a consequence of the disease (17). CRC, a high-risk disease, is often diagnosed at an advanced stage and has a high incidence among individuals aged 60–80 years (18). Moreover, with recent economic development, environmental pollution, sedentary lifestyles, and increased fast food consumption, the age of morbidity is gradually reducing (19); therefore, early diagnosis and treatment are vital for improving the overall survival rate of patients with CRC. Peripheral blood, a substitute for tissue biopsy, is convenient to collect and easy to analyze for biomarker detection (20).

Overexpression of CXCL7 can enhance the activity of the Ras/Raf/mitogen-activated protein kinase (MAPK) and PI3K/AKT/mTOR signaling pathways by binding to chemokine receptor CXCR1/CXCR2 (15, 21–23). Thus, CXCL7 has been reported as a potential biomarker in several cancers (21, 24); for example, CXCL7 mRNA expression levels in peripheral blood samples appear to be an attractive biomarker for renal cell carcinoma (24). In our study, serum levels of CXCL7 were found to be higher in the CRC group than in controls. In addition, there were higher CXCL7 levels in samples from patients with tumors at each TNM stage, relative to controls. Importantly, ROC curve analysis generated high AUC values in both the training and validation sets, providing support for the potential of serum CXCL7 expression as a biomarker for CRC diagnosis. We also conducted diagnostic tests using samples from all patients with CRC and controls. A combination of all four serum biomarkers generated the highest AUC value, 0.933, relative to any single biomarker. Patients with stage I–II tumors alone were included in a separate ROC analysis. CXCL7 had a higher AUC value (0.823) than the other three biomarkers. Similarly, the AUC value for the combined biomarkers had the highest diagnostic value (0.904).

A previous study explored the value of testing for five microRNAs for CRC diagnosis (25). The results of AUC analysis revealed that the sensitivity and specificity were increased compared with individual arbitrary biomarkers, illustrating that appropriate combinations of tumor biomarkers can exhibit greater efficiency for cancer diagnosis. Several studies have focused on diagnostic efficiency biomarker panels (26, 27), with satisfactory results. Thus, this approach can facilitate superior diagnostic methods, which could compensate for the inadequacy of screening for CXCL7 alone, thereby increasing the value of CXCL7 as a biomarker. Importantly, the results of our study are consistent with those of previous investigations, indicating that combining serum biomarkers can lead to more efficient diagnosis of CRC (28). According to the results discussed above, although single biomarkers may be of limited use in CRC diagnosis, joint analysis of several tumor markers can ameliorate these shortcomings to some extent, resulting in improved CRC diagnosis. Notably, similar conclusions have been reached for other tumor types (29). In this study, we first generated ROC curves, cut-off values, and Youden index graphs for each diagnostic test. These three graphs together clearly illustrate the results. As shown in Table 1, we found that CXCL7 concentration differed significantly between males and females; therefore, we conducted sex subgroup analysis, which demonstrated that the four elevated serum biomarkers had superior AUC values of 0.947 in the male group and 0.919 in the female group, and also exhibited high accuracy.

This study has several limitations. First, we evaluated four factors as candidate biomarkers, which is a somewhat small number. To solve this problem, in future studies we will select different serum biomarkers, collected at different time points, such as epidermal growth factor, angiogenic factors, and inflammatory factors. Second, the number of patients in our study was a little low, given that the patients with CRC enrolled in our study were all from one hospital. To address this, we will conduct a multicenter and multi-level study with a more diverse patient pool for future analyses (30). Third, this retrospective study had a cross-sectional design, rather than being a continuous tracking investigation; therefore, we will conduct a cohort study to collect dynamic information on the condition of patients over a period of time, to generate more accurate and reliable information, and avoid differences caused by the effects of timing. Further, it was not clear why serum levels of CXCL7 were higher in the male than in the female group. We speculate that CXCL7 may be influenced or regulated by key factors such as MEIS1 (Myeloid Ecotropic Viral Integration Site 1) and Galectin-3 (31, 32). If possible, we plan to measure these relevant indices in the future.

In conclusion, to the best of our knowledge, our study is the first to use blood serum CXCL7 levels for the diagnosis of CRC. Our results suggest that serum CXCL7 levels could be used as an auxiliary biomarker for CRC diagnosis. Notably, our logistic regression analysis of combined biomarkers resulted in superior diagnostic efficiency compared with each biomarker individually.

## Data Availability Statement

The raw data supporting the conclusions of this manuscript will be made available by the authors, without undue reservation, to any qualified researcher.

## Ethics Statement

This study was carried out in accordance with the recommendations of Hospital Ethics Committee in the Affiliated Hospital of Jiangnan University, and with written informed consent from all subjects. All subjects gave written informed consent in accordance with the Declaration of Helsinki. The protocol was approved by Hospital Ethics Committee in the Affiliated Hospital of Jiangnan University.

## Author Contributions

YM, XQ, and LL conceived the study. LL, LZ, and YT designed the experiments. LL, LZ, YL, GD, and XQ performed the experiments and analyzed the data. LL, LZ, YY, TZ, and DH helped with manuscript drafting and revision. All authors read and approved the final manuscript.

### Conflict of Interest

The authors declare that the research was conducted in the absence of any commercial or financial relationships that could be construed as a potential conflict of interest.

## Supplementary Material

Supplementary Figure 1 Correlation between the level of CXCL7 and those of tumor associated antigens in the CRC group. **(A–C)** Correlations between CXCL7 and CEA, CA125, and CA19-9 in samples from all CRC patients. **(D–F)** Correlations between CXCL7 and CEA, CA125, and CA19-9 in samples from patients with stage I tumors. **(G–I)** Correlations between CXCL7 and CEA, CA125, and CA19-9 in samples from patients with stage II tumors. **(J–L)** Correlation between CXCL7 and CEA, CA125, and CA19-9 in samples from patients with stage III tumors. **(M–O)** Correlation between CXCL7 and CEA, CA125, and CA19-9 in samples from patients with stage IV tumors.

Supplementary Table 1 Serum concentrations of CXCL7 and tumor associated antigens (CEA, CA125 and CA19-9).

Supplementary Table 2 Characteristics of the training and validation sets used for diagnostic testing.

Supplementary Table 3 Diagnostic efficiency of CXCL7 determined by ROC curve analysis.

## Abbreviations

CRC: colorectal cancer
CEA: carcinoembryonic antigen
CA19-9: carbohydrate antigen 19-9
CA125: carbohydrate antigen 125
ELISA: enzyme-linked immunosorbent assay
M: Median
Q: quartile
ROC: receiver operating characteristic
AUC: area under the ROC curve
95% CI: 95% confidence interval
TNM: tumor-node-metastasis
T: depth of tumor invasion
N: nodal involvement
M: distant metastasis
IQR: interquartile range
OR: odds ratio.
