# Supplementary figures and images for: Serum Chemokine CXCL7 as a Diagnostic Biomarker for Colorectal Cancer
Source: Front Oncol. 2019 Oct 9;9:921. doi: 10.3389/fonc.2019.00921 (PMC6794610; doi:10.3389/fonc.2019.00921)

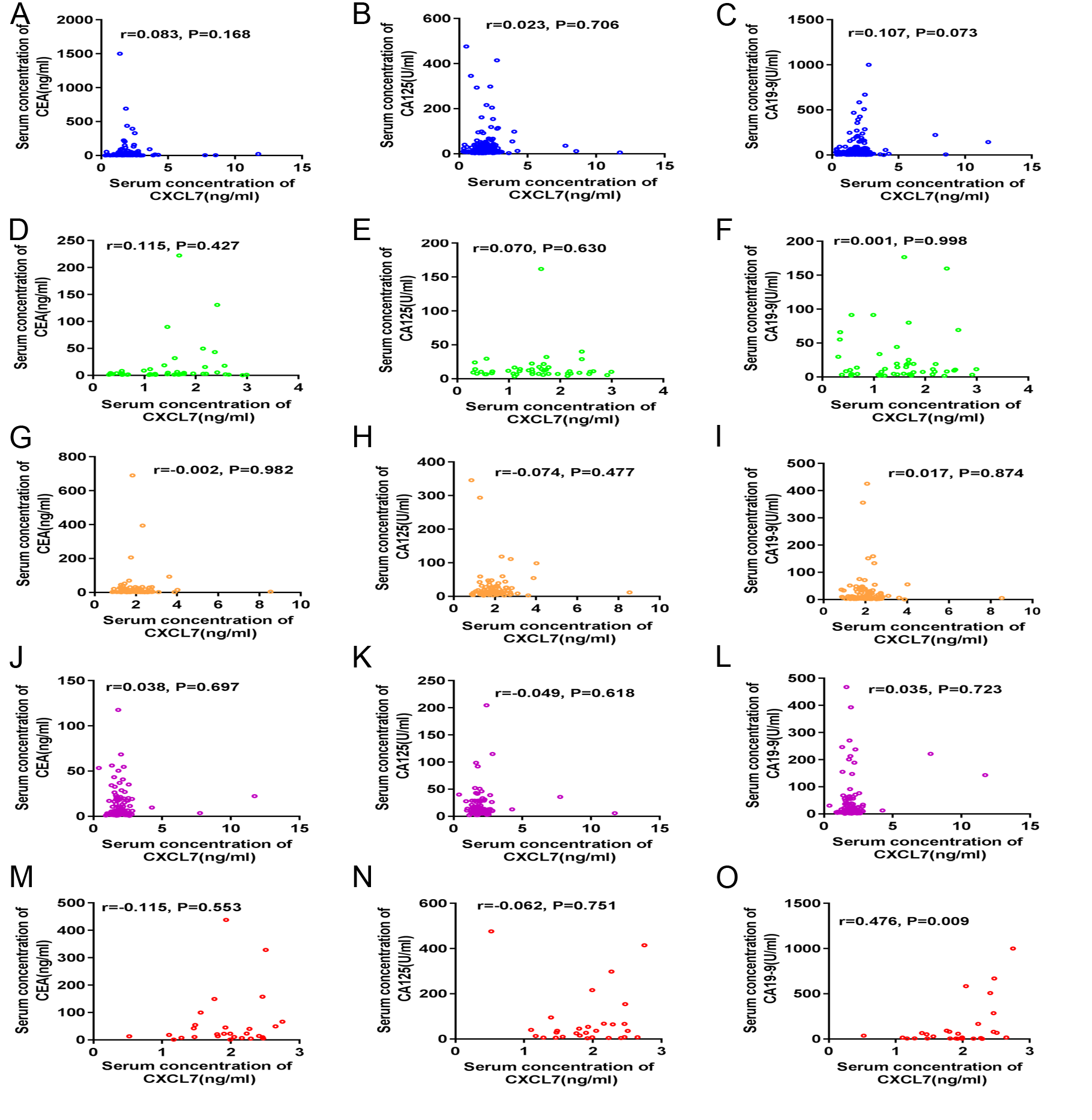

Supplement: Supplementary Figure 1 — Correlation between the level of CXCL7 and those of tumor associated antigens in the CRC group. (A–C) Correlations between CXCL7 and CEA, CA125, and CA19-9 in samples from all CRC patients. (D–F) Correlations between CXCL7 and CEA, CA125, and CA19-9 in samples from patients with stage I tumors. (G–I) Correlations between CXCL7 and CEA, CA125, and CA19-9 in samples from patients with stage II tumors. (J–L) Correlation between CXCL7 and CEA, CA125, and CA19-9 in samples from patients with stage III tumors. (M–O) Correlation between CXCL7 and CEA, CA125, and CA19-9 in samples from patients with stage IV tumors. [file Image_1.TIF]
